# Interplay between RNA Viruses and Promyelocytic Leukemia Nuclear Bodies

**DOI:** 10.3390/vetsci8040057

**Published:** 2021-03-31

**Authors:** Sabari Nath Neerukonda

**Affiliations:** Department of Animal and Food and Sciences, University of Delaware, Newark, DE 19716, USA; nnvsnath@udel.edu

**Keywords:** promyelocytic leukemia, nuclear bodies, RNA virus, small ubiquitin modifier, innate immunity, proteasome

## Abstract

Promyelocytic leukemia nuclear bodies (PML NBs) are nuclear membrane-less sub structures that play a critical role in diverse cellular pathways including cell proliferation, DNA damage, apoptosis, transcriptional regulation, stem cell renewal, alternative lengthening of telomeres, chromatin organization, epigenetic regulation, protein turnover, autophagy, intrinsic and innate antiviral immunity. While intrinsic and innate immune functions of PML NBs or PML NB core proteins are well defined in the context of nuclear replicating DNA viruses, several studies also confirm their substantial roles in the context of RNA viruses. In the present review, antiviral activities of PML NBs or its core proteins on diverse RNA viruses that replicate in cytoplasm or the nucleus were discussed. In addition, viral counter mechanisms that reorganize PML NBs, and specifically how viruses usurp PML NB functions in order to create a cellular environment favorable for replication and pathogenesis, are also discussed.

## 1. Introduction

**Figure 1:**
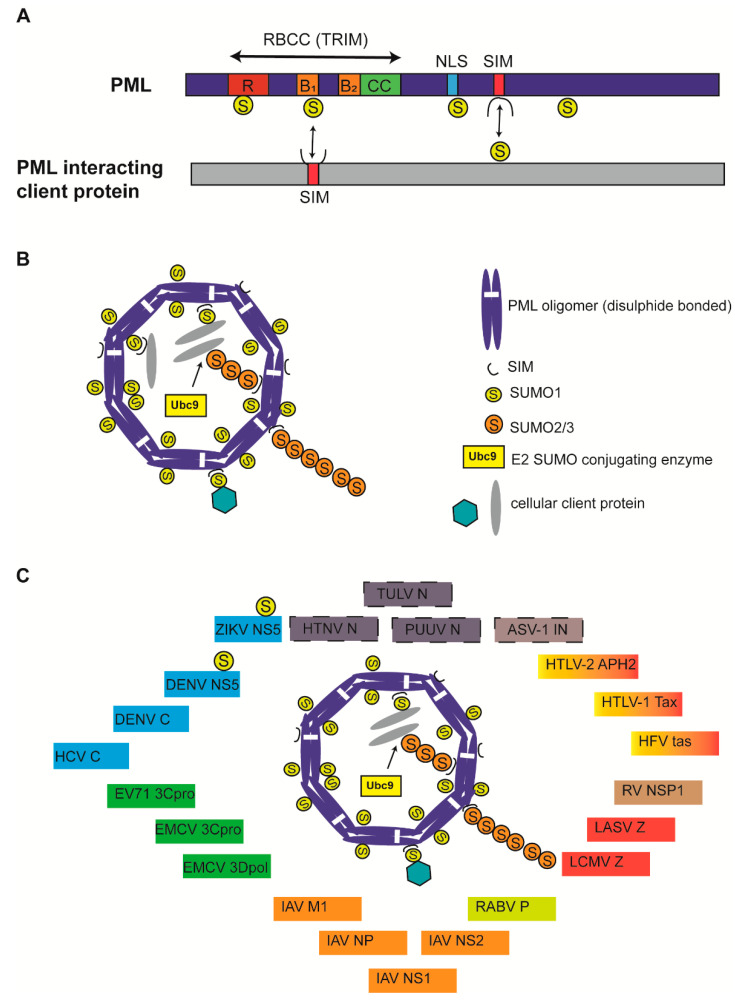
Structure of Promyelocytic leukemia (PML) protein and PML nuclear bodies (NBs). (**A**) All PML isoforms possess a conserved RBCC/TRIM motif in the N-terminus and a variable C-terminus generated due to alternative splicing. SUMOylation sites (K65, K160, K490, and K616) are indicated. Nuclear localization signal (NLS) and SUMO-interacting motifs (SIM) are indicated. PML binding with client protein via SUMO-SIM interaction and vice versa is indicated by double-edged arrows. PML protein is not drawn to scale. (**B**) Canonical PML NBs are made of an outer shell that is formed by PML oligomers that are covalently crosslinked by disulphide bonds and display non-covalent RBCC interactions. Ubc9 recruitment and SUMOylation of PML results in PML NB stabilization and client protein recruitment via client SIM-SUMOylated PML interactions. SUMO1 modifications are mainly present on the outer shell, whereas SUMO2/3 modifications in the shell also protrude into PML NB interior. PML NB is simplified for understanding and does not reflect several core PML components, including Daxx and Sp100. (**C**) Viral proteins are known to colocalize with PML NBs. Viral proteins known to interact with PML core components but undetermined for PML NB colocalization were identified with a dashed border. Viral proteins belonging to viruses taxonomically grouped in one family are identified by one color. ZIKV: Zika virus; DENV: Dengue virus; HCV: Hepatitis C virus; EV71: Enterovirus 71; EMCV: Encephalomyocarditis virus; IAV: Influenza A virus; RABV: Rabies virus; LCMV: Lymphocytic choriomeningitis virus; LASV: Lassa virus; RV: Rotavirus; HFV: Human foamy virus; HTLV: Human T-cell lymphotropic virus; ASV: Avian sarcoma virus; PUUV: Puumula virus; HTNV: Hantaan virus; TULV: Tula virus; NS or NSP: Nonstructural protein; C: Core; 3C^pro^: 3C protease; 3D^pol^: 3D polymerase; M1: matrix; NP or N: Nucleoprotein; P: Phosphoprotein; Z: Zinc finger protein; IN: integrase.

Promyelocytic leukemia (PML) nuclear bodies (NBs) are observed as 5–30 membrane-less, nuclear matrix-associated, interchromatin speckles with a diameter of 0.1–2 µm in a majority of mammalian cell types [1]. Depending upon the cell type, cell cycle phase, and the physiological state, PML NB size, number and localization can vary [2]. The main scaffold protein of PML NBs is the PML protein, which belongs to the TRIM (TRIpartite Motif) family of proteins that are characterized by the presence of a highly conserved RBCC motif made of tandemly arranged RING finger domain (R), cysteine-histidine rich B box domains (B1/B2) and an alpha-helical coiled coil domain (CC) (Figure 1A) [2,3]. Alternative mRNA splicing generates 7 different PML isoforms (I-VII) that differ in C-terminus but contain identical RBCC motifs in the N-terminus [4,5]. Isoforms (e.g., PML-VII) that lack C-terminal nuclear localization signal (NLS) remain cytoplasmic [4]. PML is subject to multiple post-translational modifications including SUMOylation, the covalent attachment of a 11 kDa small ubiquitin-like modifier (SUMO) proteins to a site-specific lysine amino acid (aa) residue. Four SUMO protein isoforms, SUMO-1, -2, -3 and -5, have been identified to be conjugated to PML at different lysines (K) with distinct functional consequences [6]. The main PML SUMOylation sites are K65, K160, and K490, although other sites such as K616 have also been reported [7,8]. PML itself is a SUMO E3 ligase [9]. PML also comprises a SUMO interacting motif (SIM) (aa. 556–562), which enables it to interact with SUMOylated PML and SUMOylated client proteins [10]. Through SUMO-SIM interactions, PML forms the main scaffold organizer of PML NBs (Figure 1B).

PML NB formation involves covalent disulphide linkage of oxidized PML monomers and non-covalent interactions between RBCC domains leading to multimeric oligomerization to form a PML NB outer shell [11,12,13]. RBCC oligomerization triggers the recruitment of Ubc9, a SUMO E2 conjugating enzyme that SUMOylates PML, leading to further PML–PML interactions via SUMO-SIM interactions [14]. Furthermore, SUMOylated PML recruits SIM-containing client proteins into an NB inner core via SUMO-SIM interactions, ultimately resulting in liquid–liquid phase separation into mature PML NBs (Figure 1B) [2]. Mature PML NBs therefore serve as SUMOylation hotspots, but can also promote other modifications such as phosphorylation, acetylation, methylation, ubiquitination, or protein degradation. PML SUMOylation is essential for mature NB formation and functions; PML mutants deficient of SUMOylation (3KR) or SIM interactions (ΔSIM) still form PML NBs exactly as wild type (WT) PML, although 3KR failed to recruit key PML NB interaction partners Sp100 and Daxx [15,16]. Mature PML NBs function in various cellular pathways, including cell proliferation, DNA damage, apoptosis, transcriptional regulation, stem cell renewal, alternative lengthening of telomeres, chromatin organization, epigenetic regulation, autophagy, intrinsic and innate antiviral immunity [2].

Type I interferons (IFNs) play a critical role in mediating innate immunity against viruses by the induction of several IFN stimulated genes (ISGs) that function at many levels of virus multiplication cycles, including entry, transcription, translation, genome replication, assembly, and release [17]. IFN production occurs upon sensing viral genomes or replication intermediates by pattern recognition receptors to initiate downstream signaling and IFN-β production [17,18]. PML-II specifically associates with transcription factors NFκB and STAT1, as well as the CBP, to facilitate transcriptional complex formation on gene promoters of IFN-β and numerous ISGs for an efficient induction of transcription [19]. PML-IV also potentiates IRF3 induction of IFN-β by sequestering Pin1 to PML NBs, therefore preventing Pin1 negative regulation of IRF3 via ubiquitin dependent degradation [20,21]. PML was found to positively regulate not only type I IFN signaling, but also type II IFN (IFN-γ) signaling [22,23,24,25]. In fact, PML NB components (PML, Sp100, Daxx) themselves are ISGs and IFN treatment promotes PML NB formation [26,27,28].

PML NBs impose intrinsic immunity against several DNA and RNA viruses by sequestering viral proteins, chromatinization of viral DNA with specific histone variants or epigenetic marks, SUMOylation of viral or cellular proteins to alter protein stability and function, and promoting IFN mediated effects, including IFN signaling, IFN-induced SUMOylation, IFN-induced apoptosis and IFN-induced antiviral effects [17,29,30]. Although numerous studies have confirmed that the nuclear replication of many DNA viruses is intimately intertwined with PML NBs, several studies point to a critical role of PML NBs in determining the outcome of cytoplasmic and nuclear replication of several RNA viruses from diverse families [29]. In the present narrative, I review the specific mechanisms of how PML NBs and NB components affect the replication of RNA viruses and how RNA viruses have in turn evolved counter mechanisms to modify PML NBs or its components to create a cellular environment conducive for viral replication (Figure 1C; Table 1). Although PML NBs are popularly known as SUMOylation hotspots and there exists substantial evidence that RNA virus infection alters the global SUMOylation status of both cellular and viral proteins, there is limited information as to whether PML NBs play a critical role in carrying out essential SUMOylation or other protein modifications [31]. 

## 2. Positive Sense Single Stranded RNA Viruses

### 2.1. Flaviviridae

The family *Flaviviridae* constitutes enveloped viruses with positive-sense single stranded RNA (ssRNA) genomes [62]. The genome encodes a single polyprotein that is cleaved into three structural proteins (core (C), premembrane (prM), and envelope (E)), and seven nonstructural (NS) proteins by viral and cellular proteases [62]. Although genome replication of flaviviruses is carried out in the cytoplasm by NS5 RNA-dependent RNA polymerase (RdRp), nuclear localization of viral proteins also occurs during infection to antagonize host antiviral response and to facilitate viral replication. *Flaviviridae* includes viruses of public health concern such as Zika virus (ZIKV), dengue (DENV), yellow fever (YFV), Japanese encephalitis virus (JEV) and Hepatitis C virus (HCV) [62].

#### 2.1.1. Zika Virus

ZIKV is a mosquito-borne flavivirus linked to a congenital Zika syndrome, characterized by fetal microcephaly, and to Guillain–Barré syndrome. ZIKV (PRVABC59 strain) undergoes persistent infection and replication in primary human brain microvascular endothelial cells (hBMECs) without cytopathology, whereas it undergoes lytic infection in neuronal progenitor cells (NPCs), trophoblasts, and other cell types by differential regulation of cytotoxic responses [32]. On the other hand, ZIKV constitutively induces and evades IFN and ISG expression in all cell types. In ZIKV-infected hBMECs, NS5 proteins, display nuclear colocalization with PML NBs, which increase in size as the infection progresses [32]. ZIKV NS5 colocalizes with SUMO-1 and STAT2 in PML NBs to disrupt SUMO-1 and STAT2 colocalization with PML, and promotes PML degradation [32]. A conserved SIM (VIDL) in the NS5 methyltransferase domain other than the bipartite NLS in RdRp domain is required for ZIKV NS5 nuclear localization and transcriptional regulation of ISG and cell cycle genes [32,63]. By regulating IFN and the cell cycle, ZIKV undergoes persistent infection and replication in hBMECs without cytopathology. Notably, SUMOylation of ZIKV NS5 is SIM-independent [32]. In NPCs, on the contrary, nuclear localization of NS5 resulted in a significant reduction in the number of PML NBs [33].

ZIKV infection of NPCs and HepG2 cells was demonstrated to induce kynurenine (Kyn), a tryptophan-derived aryl hydrocarbon receptor (AHR) ligand typically produced during inflammation by the enzymatic activity of indoleamine 2,3-dioxygenase 1 and 2 and tryptophan 2,3-dioxygenase [64]. Kyn activates AHR signaling, which favors ZIKV replication by suppressing NFκB activation, and expression of IFN and ISGs including PML. AHR signaling limited PML expression by suppressing NFκB activation, as well as by binding AHR-responsive elements in the PML promoter [64]. AHR inhibition by CH223191 inhibitor suppressed ZIKV replication, which was partially rescued upon PML knockdown [64]. Conversely, overexpression of PML-III suppressed ZIKV replication [64]. These observations identify PML-mediated restriction of ZIKV replication and ZIKV-induced activation of AHR to limit IFN, as well as PML-mediated intrinsic immunity. 

#### 2.1.2. Dengue Virus

DENV is a mosquito borne flavivirus with four DENV serotypes (DENV1–4) affecting millions of individuals each year by causing life threatening dengue hemorrhagic fever and dengue shock syndrome [34,35]. As described above for ZIKV, AHR activation was enhanced, and conversely, AHR inhibition suppressed, replication of all four serotypes of DENV in A549 cells [64]. PML silencing rendered higher virus production in A549 cells for all four DENV serotypes, whereas overexpression of PML-III or -IV reduced virus production significantly. Furthermore, a considerable reduction in the number of PML NBs was noticed in DENV-infected cells [34,35]. Specifically, DENV2 NS5 colocalized with PML-III/-IV in nuclear speckles, resulting in PML-III/-IV degradation [35]. In contrast, NS5 in DENV3-infected hBMECs displayed a uniform presence in the nucleoplasm and suppressed basal IFN and ISG expression [32]. The DENV3 NS5 SIM site is required for both NS5 SUMOylation and nuclear localization [32]. Interestingly, DENV NS5 localization changed to discrete punctate NBs when the NS5 SUMO site was mutated to K546R [32].

DENV C protein directly interacts with Daxx in the nucleus and sensitizes hepatic cells to Fas-mediated apoptosis [36]. Pro- or anti-apoptotic functions of Daxx are mediated by PML NBs [65]. In the nucleus, Daxx-induced cell death appears to be mediated by the PML-NBs, presumably via protein interactions within PML-NBs. Mechanistically, Daxx-induced cell death involves transcriptional repression of anti-apoptotic genes such as survivin/Bcl2, activation of ASK1–JNK cascade in the nucleus upon UV exposure, and transcriptional activation of proapoptotic genes via p53 activation [66,67,68]. DENV C binds the carboxyl terminus of Daxx, the same region involved in the interaction with the PML [37]. While the authors originally proposed that competitive binding between DENV C and PML with Daxx may limit Daxx-PML interaction, thereby releasing Daxx from PML NBs into the nucleoplasm, this remains to be confirmed [36]. 

#### 2.1.3. Hepatitis C Virus

HCV is among the leading causes of viral hepatitis, and chronic HCV infection is associated with liver cirrhosis and progression to hepatocellular carcinoma [39]. A fraction of HCV C protein colocalizes with PML and p53 in PML NBs of human hepatoma cells. HCV C interacts with PML-IV, which serves as a transcriptional coactivator of p53. C protein abolishes the PML-IV coactivator function by inhibiting p53 post translational modifications (S15 phosphorylation and K382 acetylation), thus repressing p53 pro-apoptotic targets, CD95 (Fas), PIG3, and bax [39]. 

PML deficiency in HCV-transgenic (HCV_tg_) PML-deficient (PML^−/−^) mice enhanced susceptibility to tumor induction by diethylnitrosamine (DEN)/phenobarbital (PB) treatment compared to WT, HCV_tg_, and PML^−/−^ mice groups by enhancing hepatocyte proliferation [38]. In the absence of DEN/PB induction, 40% of HCV_tg_ PML^−/−^ mice maintained for long-term duration also formed spontaneous tumors with greater hepatocyte proliferation, whereas no liver lesions were observed among the other groups [69]. A significant downregulation of PML and NFκB target genes associated with tumor suppressive functions (*RASSF6, NLRP12)* was observed in HCV_tg_ PML^−/−^ mice [69]. These mechanisms highlight the potential tumor-suppressive functions of PML in HCV-associated carcinogenesis. PML activities are usurped by HCV to facilitate the replication in a genotype-specific manner as shown by increased replicon activity of genotypes 1b in the presence of all PML isoforms and 2a (PML-I/-III/-V isoforms), but not 3a [70].

### 2.2. Picornaviridae

*Picornaviridae* constitutes a family of non-enveloped viruses with a positive-sense ssRNA genome encoding a single polyprotein that is co- and post-translationally processed by viral proteases (2A^pro^ and 3C^pro^) into structural virion proteins and non-structural proteins [71]. Among nonstructural proteins, the RdRp (3D polymerase [3D^pol^]) facilitates the replication of viral genome in the cytoplasm, although several non-structural proteins including 2A^pro^, 3C^pro^, 3D^pol^ and 3CD^pol^ were found in the nucleus to deregulate several host cellular processes [72,73].

#### 2.2.1. Polio Virus

Poliovirus (PV) is an etiological agent of paralytic poliomyelitis [71]. PML-III expression exerts an anti-viral effect towards PV in a p53 dependent manner [40]. PV infection induces an early ERK activation, which triggered PML phosphorylation and subsequent SUMOylation, leading to an increase in PML NB size. Eventually, p53 is recruited to PML NBs and phosphorylated at S15 in a PML-dependent manner followed by transcriptional activation of p53 target genes, Mdm2 and Noxa, resulting in apoptosis of infected cells [40]. Knockdown (KD) of p53 or PML by siRNA results in a higher poliovirus replication, indicating that both PML and p53 exert an antiviral effect [40]. However, an antiviral effect of PML and p53 is transient and is countered by PV-induced degradation of p53 in a proteasome- and MDM2-dependent manner [40]. 

#### 2.2.2. Enterovirus 71

Enterovirus 71 (EV71) is the common cause of hand, foot, and mouth disease [41]. PML functions as a restriction factor for EV71 replication. Both viral yields and protein production were enhanced upon PML KD or deficiency [41]. Conversely, overexpression of PML-III and -IV isoforms, but not other isoforms, reduced viral yields and protein production. PML inhibited EV71 replication by a negative regulation of autophagy. Despite the intrinsic anti-viral activity towards EV71 replication, PML also mediated the antiviral activity of IFN-β and this activity was abrogated in the PML^−/−^ HeLa cells. As a viral countermeasure, EV71 has evolved to significantly reduce the number of PML NBs and PML-III/-IV protein levels early upon infection, which further reduced as infection progressed [41]. Mechanistically, EV71 3C^pro^ mediated cleavage of PML-I, -II, -III, -IV, -V and -VI causes this reduction [41]. On the other hand, 3C^pro^ 45–52 aa. residue stretch was found to interact with Ubc9 to become SUMOylated at K52 [74]. 3C^pro^ SUMOylation was found to target it to SUMOylation-dependent ubiquitination and degradation, thus reducing protein stability and reduced protease activity in vitro [74]. A SUMOylation mutant (K52R) virus displayed higher replication, enhanced apoptosis, and enhanced intestinal and neurological damage in mice compared to WT virus. SUMO modification thus serves as a host cell defense mechanism designed to ameliorate EV71-induced pathogenesis [74]. Since PML NBs are known SUMOylation hot spots with Ubc9 as a PML NB component, whether 3C^pro^ SUMOylation occurs in PML NBs remains to be investigated.

#### 2.2.3. Encephalomyocarditis Virus

Encephalomyocarditis Virus (EMCV) is the prototype of the *cardiovirus* genus [71]. EMCV infection of PML^−/−^ mouse embryonic fibroblasts (MEFs) resulted in higher viral replication and protein production. Conversely, over expression of PML-III or -IV reduced viral replication and protein production in a SUMOylation dependent manner, with PML-IV displaying substantial antiviral effect [42]. Although, EMCV induced early PML NB formation in PML-III expressing CHO cells, SUMOylated PML-III underwent degradation via proteasome during later stages of replication. Specifically, viral 3C^pro^ colocalized with PML NBs and induced PML-III degradation during final stages of infection [42]. Furthermore, PML-IV or -IVa (lacks exon 5) interacted with 3D^pol^ via C-terminus and sequestered it within PML NBs, potentially preventing its cytoplasmic replication functions [43]. 

## 3. Negative Sense Single Stranded RNA Viruses

### 3.1. Orthomyxoviridae

The family *Orthomyxoviridae* constitutes a family of enveloped viruses with 6–8 segments of negative sense ssRNA genomes. They comprise influenza viruses of mammals and birds [75]. Influenza viruses replicate their genomes in the nucleus and several encoded proteins display wide range of innate immune antagonistic functions [75].

#### Influenza A Virus

Influenza A virus (IAV) is a constant threat to human and veterinary health worldwide. While avian reservoirs harbor most of the influenza subtypes, only H1, H2, H3, H5, H7, H9, and H10 subtypes have been found in humans, with the former 6 subtypes associated with human disease [76,77]. Overexpression of PML-III and -IV or -VI was demonstrated to reduce replication of H1 (WSN) and H3 (A/Hokkaido/92/99) influenza viruses, respectively [78,79]. In contrast, PML deficiency had no effect on replication of FPV-B, a mammalian cell-adapted variant of H7N7 IAV (A/FPVDobson/34) in PML^−/−^ MEFs. Mx1 GTPase is an ISG and IAV resistance factor that was found partially overlapping or juxtaposed to PML NBs and interacts with PML NB components including Daxx, Sp100, SUMO1, BLM, TOPORS and PKM/HIPK-2 [80,81]. However, PML NBs were found to be dispensable for Mx1 antiviral activity as WT and PML^−/−^ MEFs transduced with Mx1 displayed comparable levels of replication [81]. Finally, PML displayed strain-specific antiviral effect on IAV replication [82]. While PR8 (H1N1) and ST364 (H3N2) strains underwent enhanced replication upon PML KD, ST1233 (H1N1), Qa199 (H9N2), and Ph2246 (H9N2) strains had no replication differences upon PML KD. On the other hand, PML-VI overexpression displayed antiviral effect towards PR8 (H1N1) replication but not remaining IAV strains, indicating isoform-specific antiviral effects of PML [82]. IAV matrix protein (M1), nucleoprotein (NP), and the nonstructural proteins, NS1 and NS2 (NEP) have been shown to associate with PML NBs, although the functional significance of these associations remains unclear [44,45,46].

### 3.2. Pneumoviridae

*Pneumoviridae* constitutes a family of enveloped negative-sense ssRNA viruses that infect humans, mouse, cattle, and birds [83]. A significant member of the family that affects humans is the respiratory syncytial virus (RSV) which causes acute lower respiratory tract infections associated with bronchiolitis and pneumonia in children < 5 years [83]. Although RSV has a cytoplasmic replication cycle, its matrix (M) protein has been found inside the nucleus of infected cells suggesting nuclear alteration during infection [84].

#### Respiratory Syncitial Virus

Reactive oxygen species (ROS) produced by epithelial and inflammatory cells, and subsequent oxidative stress, and downregulation of anti-oxidant enzyme (AOE) genes is implicated in RSV-induced lung injury [47]. AOE gene expression is induced by NRF2, a basic leucine zipper transcription factor that is normally inhibited in the cytosol by KEAP1 (Kelch-like-ECH Associated Protein 1) binding [47]. KEAP1 binding renders NRF2 inactive by targeting it for degradation by the ubiquitin-proteasome pathway. However, when ROS are generated, KEAP1 undergoes a conformational change and releases NRF2, which translocates to nucleus to bind to antioxidant response elements (ARE) or MAF recognition elements (MARE) and promotes AOE gene transcription [47]. KEAP1 independent pathways controlling NRF2 degradation also exist such as RNF4 dependent degradation of SUMOylated NRF2. RNF4 is SUMO-specific E3 ubiquitin ligase that is localized to PML NBs upon prolonged ROS exposure. RSV infection in A549 cells promotes PML NB formation in an IFN-dependent manner where NRF2 interacts with PML, gets SUMOylated, and is targeted for degradation by RNF4 [47]. On the other hand, RSV infection in A549 cells was also demonstrated to dissolve PML NBs and relocalize NB components, PML and Sp100 to cytoplasm [48].

### 3.3. Rhabdoviridae

The family *Rhabdoviridae* constitutes enveloped negative-sense ssRNA viruses. The prototype members of this family include vesicular stomatitis virus (VSV) and rabies virus (RABV) [85]. *Rhabdoviridae* members replicate in cytoplasm by means of nucleoprotein (N), phosphoprotein (P), and RNA-dependent RNA polymerase (L) proteins [85].

#### 3.3.1. Vesicular Stomatitis Virus

PML^−/−^ MEFs and PML^−/−^ mice displayed higher sensitivity to VSV replication and infection compared to their WT counterparts [20,79]. Among PML isoforms, both PML-III and -IV displayed antiviral activity, with PML-IV having a predominant antiviral effect [20]. While PML-III acted in an IFN-independent manner, PML-IV displayed an early IFN-independent activity targeting VSV replication and a later activation of innate immune pathways, leading to an enhanced type I IFN synthesis [20]. Antiviral activities of PML-IV were SUMOylation-dependent. Specifically, PML-IV induced potent IRF3 activation and IFN-β induction by sequestering endogenous Pin-1 in PML NBs. Pin1 is known to interact with phosphorylated IRF3 and to promote its ubiquitin-mediated proteasomal degradation [20,21]. Pin1 interaction with PML-IV and subsequent colocalization in PML NBs resulted in sustained IRF3 activation and higher IFN-β induction upon poly I:C treatment or infection with a variety of DNA or RNA viruses including Sendai virus, EMCV, human T-cell lymphotrophic virus 1, IAV or vaccinia virus [20]. These mechanisms highlight a general role of PML-IV in IFN induction apart from its intrinsic antiviral activity. 

#### 3.3.2. Rabies Virus

RABV infection of PML^−/−^ MEFs resulted in higher viral replication and reduced IFN sensitivity, indicating a PML anti-viral role during RABV infection [49]. Specifically, over expression of PML-IV or -IVa isoforms inhibited viral mRNA and protein synthesis, leading to a reduction in viral replication in a PML SUMOylation-dependent manner [49]. PML-IV interacts with RABV P protein to counteract its involvement in viral transcription and other immune evasion mechanisms, producing an antiviral effect [86]. Since RABV infection prevents IFN induction in infected cells, the anti-viral effect of PML-IV is rather intrinsic. In addition, PML-III (via RING domain) also interacts with the RABV P and its truncated isoforms (P2 and P3) in transfected and infected cells. While P protein delocalized PML to a cytoplasm, P3 protein displayed a speckled appearance in nucleus where it reorganized PML NBs to a greater NB size [86]. PML-III and remaining PML isoforms (PML-I-VI and -VIIb), however, displayed no effect on viral replication and yield upon overexpression [49].

### 3.4. Arenaviridae

The family *Arenaviridae* comprises a large number of mammalian arenaviruses that are enveloped and comprise a negative sense of ssRNA genomes in two ambisense segments (small and large) [87]. They are classified into two major groups: the Old World and the New World arenaviruses [88]. The Old World group includes the prototype lymphocytic choriomeningitis virus (LCMV) that bears significance in transplantation and pediatric human medicine. The highly pathogenic Lassa virus (LASV) causes a severe viral hemorrhagic fever with high mortality in Western Africa. Highly pathogenic arenavirus transmission to humans occurs mainly via reservoirs by inhalation of aerosolized rodent excreta, skin abrasions, or ingestion of contaminated food [87].

#### Lymphocytic Choriomeningitis Virus

LCMV infection of PML^−/−^ MEFs resulted in reduced Type I or II IFN sensitivity and higher virus yields [89,90]. Furthermore, PML^−/−^ 129Sv mice displayed higher viral titers and severe central nervous system or liver pathology compared to WT mice. Notably, PML deficiency had no effect on cytotoxic T cell (CTL) responses but an early innate immune response that limits primary viral replication [90]. In line with the PML antiviral role against arenaviruses in general, LCMV and LASV Z proteins in transfected cells were found to interact with PML and redirect it to punctate bodies in the cytoplasm. In the cytoplasm, both proteins interacted with the eukaryotic initiation factor 4E (eIF4E), reducing its affinity for the N7-methyl guanosine cap of mRNA by over 100-fold, resulting in host translational repression [50,51]. LCMV infection of NIH-3T3 cells also results in delocalization of nuclear PML to predominantly cytoplasmic punctate bodies [50]. 

Arenaviral Z proteins contain a highly conserved RING finger domain that co-ordinates two zinc atoms. Remarkably, disulfide- (NSC20625, AT-2) and azo-based (ADA) compounds induced metal-ion ejection and multimeric aggregation of Z proteins, with the consequent loss of its native structure and stability [91]. However, NSC20625 displayed no effect of cellular RING proteins, including PML and, moreover, restored PML distribution from a diffuse-cytoplasmic pattern to native punctate, discrete NBs in LCMV-infected HepG2 cells [91,92]. Additionally, the above compounds also destroyed virus infectivity by potentially altering the virion Z protein structure, which constitutes the matrix protein of virion lipid envelope needed for entry [91]. In particular, ADA was much less effective than the disulfide compounds in reducing virus yields from infected cells [91]. 

### 3.5. Bunyaviridae

*Bunyaviridae* is a family of enveloped negative sense ssRNA viruses that are usually found in arthropods, rodents and bats [93]. Occasional transmission to humans can cause deadly hemorrhagic fever and pulmonary syndrome outbreaks. It includes 5 genera: Phlebovirus, Nairovirus, Hantavirus, Orthobunyavirus, and Tospovirus [93]. 

Puumala virus nucleoprotein (N) can interact with the nuclear Daxx, which is a known PML NB component and a Fas-mediated apoptosis enhancer [55]. Kaukinen et al. hypothesized that the Daxx binding of hantaviral N proteins in the cytoplasm could prevent Daxx-mediated transcriptional repression in the nucleus and trigger the Fas–apoptosis pathway in the cytoplasm of hantavirus-infected cells [94]. Hantaviruses, Andes virus and Hantaan virus triggered PML NB formation upon replication in human umblical vein endothelial cells (HUVECs) [52]. Although PML NBs contained Sp100, it lacked other NB components Daxx and SUMO1 [52]. This led the authors to hypothesize this as a potential mechanism by which the virus prevents Daxx-mediated apoptosis. Finally, Tula and Hantaan virus N proteins were observed to interact with SUMO-1 and Ubc9 in the perinuclear region, although whether N proteins relocate Ubc9 and SUMO-1 from PML NBs is unclear [53,54].

## 4. Double Stranded RNA Viruses

### 4.1. Reoviridae

Viruses of the Reoviridae family are non-enveloped icosahedral viruses containing 9 to 12 segments of linear double-stranded (ds) RNA [95]. Although reovirus replication occurs in the cytoplasm, several viral proteins have been detected in the cell nucleus. 

#### Rotavirus

Rotaviruses are a major cause of severe gastroenteritis in infants and young children. Rotaviruses are three-layered, non-enveloped viral particles comprising 11 linear dsRNA genome segments encoding each of the six structural (VP1 to VP4, VP6, and VP7) and six nonstructural (NSP1 to NSP6) proteins [96]. Although rotavirus replication cycle and assembly take place in the cytoplasmic inclusions known as viroplasms, and by endoplasmic reticulum (ER) membrane budding respectively, NSP1 was found to localize in both cytoplasm and nucleus. NSP1 is a multifunctional protein that also functions as a substrate adaptor for Cullin RING ligases to target several proteins in IFN induction pathway for ubiquitin-dependent proteasome degradation [96,97]. NSP1 of several rotavirus groups encode a highly conserved N terminal RING finger essential for its functions and a diverse C-terminus [96,97]. NSP1 displayed strain specific effects on PML NBs. NSP1 displayed PML colocalization and speckled appearance in OSU-strain infected cells, whereas NSP1 of SA11–4F or -5S strains displayed diffuse nuclear localization and lacked PML NB colocalization [56]. While infection with OSU strain enhanced the area of PML NBs as oblong structures, infection with other SA11–4F-like rotaviruses (SA11-L2, SNF, and SRF) and OSU-like virus (SDF) resulted in a substantial reduction in the number of PML NBs compared to uninfected cells [56]. Despite the above effects on PML area and number, PML KD had no significant effect on the SA11–4F and OSU viral yields in HaCaT cells, which in general are highly permissive for rotavirus replication and therefore, any PML NB antiviral effects may have been masked [56].

## 5. Reverse-Transcribing Viruses

### 5.1. Retroviridae

Retroviruses are characterized as enveloped viruses with positive sense ssRNA genomes, which, upon infection, are reverse-transcribed into DNA in order to be integrated into the host chromosome [98]. The integrated provirus utilizes the promoter elements in the 5’ long terminal repeat (LTR) to initiate transcription to give rise to the unspliced full length mRNA that will serve as genomic RNA to be packaged into virions or a source of viral proteins by translation [98].

#### 5.1.1. Human Foamy Virus

Foamy viruses (FVs) are complex animal retroviruses with a 5’ cap and a 3’ poly-A tail on their genomes. The genome is flanked by LTRs and encodes structural or enzymatic genes (*gag*, *pol*, *env*) and ancillary genes (*bet* and *tas*) [57]. PML, via its RING finger, directly interacts with Tas and interferes with Tas-induced transactivation of human FV (HFV) LTR, thus causing transcriptional repression and HFV replication inhibition [57]. This activity of PML is independent of SUMOylation as PML 3KR also displayed similar transcriptional repression activity and HFV replication inhibition [57]. IFN-mediated anti-viral effect on HFV was completely abrogated in PML^−/−^ MEFs, highlighting the predominant role of PML in mediating both intrinsic and IFN antiviral activity against HFV [57].

A subsequent study has, however, demonstrated no correlation between endogenous PML levels and HFV replication [99]. Cell lines that exhibited HFV lytic replication displayed identical PML expression levels to those that supported latent infection. Furthermore, HFV replication proceeded in HT1080 cells, expressing higher levels of PML and endogenous PML lacked colocalization with Tas in the presence or absence of IFN [99]. Finally, PML had no role in the maintenance and reactivation from latency, although IFN treatment abolished reactivation from latency [99]. The findings from the above two studies are conflicting and differences in cell types (human vs mouse), and the experimental approach (exogenous overexpression vs endogenous PML) might account for these differences.

#### 5.1.2. Human Immunodeficiency Virus 1

PML restricts HIV-1 in MEFs during early post-entry stages of infection and contributes to transcriptional silencing of integrated provirus [100,101,102]. Furthermore, human PML isoforms I, II, IV, and VI rescued PML restriction in PML^−/−^ MEFs. Type I IFN restriction of HIV-1 in MEFs is partly mediated by PML. PML restriction of HIV-1 is, however, cell-type specific. In human cells, PML KD has either no effect or a modest effect on HIV-1 infectivity. PML restricts HIV-1 infectivity in human foreskin fibroblasts by 2- to 3-fold, possibly through stimulation of ISG expression by direct association with ISG promoters [23,101]. In several other human cell types investigated, including Jurkat, THP-1, HeLa, and TE671 cells, Type I IFN restriction of HIV-1, was found to be PML-independent. Similarly, PML neither had an intrinsic antiviral activity against HIV-1, nor a transcriptional silencing activity in these cell types [58]. On the other hand, Type I IFN restriction of simian immunodeficiency virus (SIV_mac_) was significantly greater in the presence of PML in Jurkat cells [58].

#### 5.1.3. Human T-Cell Lymphotropic Virus 1 and 2

Human T-cell lymphotropic virus type I (HTLV-1) is a prototypic oncoretrovirus that is etiologically linked to adult T-cell leukemia/lymphoma (ATL), an aggressive malignancy that involves CD4+ T cell transformation upon infection [103]. The viral oncoprotein Tax and HBZ (HTLV-1 basic leucine zipper) cooperatively facilitate T-cell transformation through deregulation of multiple cellular pathways. Continued expression of Tax is essential for the survival of HTLV-1-transformed cells in vitro. Treatment of ATL-derived or HTLV-1-transformed cells with an arsenic+IFN combination triggers PML NB formation and recruitment of Tax, which undergoes SUMOylation through SUMO2/3 [103]. SUMOylated Tax is targeted for ubiquitination by RNF4 SUMO E3 ubiquitin ligase in PML NBs and ubiquitin proteasomal degradation [103]. In line with this, in chronic ATL patients, a combination of arsenic, IFN, and the nucleotide analog zidovudine promoted complete and durable clinical remissions [59].

In contrast to HTLV-1, HTLV-2 preferentially infects CD8+ T cell infection and most infected carriers remain asymptomatic [60]. As with HTLV-1 HBZ, HTLV-2 encodes an Antisense Protein of HTLV-2, termed APH-2 [60]. Although aph-2 mRNA is detected, APH-2 protein is barely detectable in vitro. APH-2 is efficiently targeted to PML NBs via its C-terminal LXXLL domain, where it is SUMO1/2/3-modified and targeted for proteasomal degradation [60]. In contrast to HTLV-1 Tax, HTLV-2 Tax protein is barely SUMOylated, highlighting potential roles of PML NBs and SUMOylation in determining oncoprotein stability and tumorigenesis [60].

#### 5.1.4. Avian Sarcoma Virus

In avian sarcoma virus (ASV-1)-infected mammalian cells, PML NB component Daxx was known to bind viral integrase, and associate with viral DNA to cause transcriptional repression, potentially by the recruitment of histone deacetylases [61]. This Daxx-mediated restriction is intrinsic and is redundant to IFN mediated suppression of viral replication [104]. Whether Daxx-mediated restriction is PML NB dependent is still unclear.

## 6. PML Targeted Antiviral Therapy

The PML gene was first discovered in an acute promyelocytic leukemia (APL) as part of PML-RARα fusion oncoprotein. The PML-RARα fusion disrupts PML NBs biogenesis [105]. In APL patients, two compounds, arsenic trioxide (ATO) and all-trans retinoic acid (ATRA), were demonstrated to directly target PML-RARα, resulting in a 95% cure rate [105]. While ATRA binds and targets RARα to reverse PML-RARα transcriptional regulation and proteasomal degradation, ATO is thought to enhance PML SUMOylation and target PML-RARα for proteasomal degradation. ATO presumably binds to zinc finger motifs in the RING and B1-box of the PML or PML-RARα oncoprotein, causing their multimerization and conformational change, followed by the recruitment of Ubc9, which SUMOylates PML or PML-RARα [106,107,108,109,110]. Furthermore, SUMOylated PML or PML-RARα may further recruit interaction partners such as Daxx, Sp100, CPB, and RNF4, which further promotes PML or PML-RARα degradation via the ubiquitin–proteasome pathway [110]. ATO-mediated restoration of PML NB biogenesis is a critical factor in ATO therapy. ATO is FDA approved for the treatment of APL in 2000 as Trisenox^TM^ [111].

Emerging evidence points to ATO-mediated therapeutic effects in the context of several viral infections and virus-induced cancers [112,113]. As described above, ATO+IFNα combination treatment has shown a synergistic effect in inducing cell cycle arrest and apoptosis in HTLV-1 transformed cells in vitro [114]. In a pilot phase II study comprising seven patients with relapsed/refractory acute or lymphomatous ATL, the ATO+IFNα combination has shown a promising efficacy [115]. ATO +IFNα combination also displayed a synergistic effect against HCV, as observed by reduced sub-genomic RNA levels in HCV replicon-containing cells. At a sub-micromolar concentration, ATO alone completely abolished HCV replication whereas IFNα alone, even at the highest concentration, only partially abolished replication [116]. The ATO antiviral effect against HCV is, however, dependent on ATO modulation of the glutathione redox system and oxidative stress, but not PML [117]. ATO also effectively reactivated viral transcription and replication in the J-Lat HIV latency cell line model and primary CD4+ T cells from chronically SIV-infected macaques and HIV-infected patients [118]. As part of the “shock and kill” strategy, a combination of ATO and anti-retroviral therapy (ART) effectively reactivated viral latent reservoirs and delayed viral rebound upon termination of ART in chronically SIV-infected rhesus macaques [118]. Several possible mechanisms for delayed viral rebound were proposed including perturbation of viral reservoir, downregulation of CD4 primary receptors and CCR5 coreceptors for SIV entry on CD4+ T cells, as well as enhancement of SIV-specific immune responses [118]. Whether these mechanisms are linked to PML NBs is unclear. ATO is therefore of translational interest to modulate PML NB functions during viral infections.

## 7. Conclusions

PML NBs emerge as significant regulators of replication of RNA viruses. On the one hand, PML NBs display an intrinsic restriction to prevent virus multiplication. On the other hand, PML NBs serve to coactivate host cellular gene expression, including innate IFN genes. Further studies are necessary to dissect how PML-NBs exert these complementary effects during viral infections. Finally, decoding viral evasion mechanisms that counteract PML NB functions will inform strategies that leverage PML NB antiviral activity to its fullest benefit.

## Figures and Tables

**Table 1:** Viral proteins interacting with Promyelocytic leukemia nuclear bodies and its components.

Virus	Viral Protein	Mechanistic Consequence	Reference
ZIKV	NS5	ZIKV persistence in human brain microvascular endothelial cells	[32]
		PML NB disruption in neuronal progenitor cells	[33]
DENV	NS5	PML NB disruption and PML degradation in A549 cells	[32,34,35]
	C	ND	[36,37]
HCV	C	Inhibition of PML coactivation of p53 transcriptional activity to facilitate hepatocellular carcinogenesis	[38,39]
PV	ND	Transient p53 transactivation of proapoptotic targets followed by PV-induced p53 degradation	[40]
EV71	ND	3C^pro^ mediated cleavage of PML-I, -II, -III, -IV, -V and -VI isoforms; PML NB disruption and PML degradation	[41]
EMCV	3C^pro^	3C^pro^ mediated cleavage of PML-III	[42]
	3D^pol^	Nuclear sequestration to prevent 3D^pol^ cytoplasmic replication function	[43]
IAV	M1, NP, NS1, and NS2	ND	[44,45,46]
RSV	ND	PML NB formation to facilitate NRF2 SUMOylation and degradation; PML dissolution	[47,48]
VSV	ND	IRF3 activation and IFN-β induction by sequestering Pin-1, a negative regulator of IRF3	[20,21]
RABV	P	Prevent viral transcription and immune evasion functions of P protein	[49]
LCMV, LASV	Z	Relocate PML to cytoplasm where PML and Z proteins interact with eIF4E to cause translational repression	[50,51]
ANDV, HTNV	ND	Non-canonical PML NB formation where NBs lack Daxx and SUMO1 components	[52]
TULV, HTNV	N	ND	[53,54]
PUMV	N	Daxx interaction; ND	[55]
RV	NSP1 (strain specific)	PML NB disruption	[56]
HFV	Tas	Repression of Tas transcriptional activity and HFV replication inhibition	[57]
HIV-1	ND	Cell specific transcriptional repression of provirus in MEFs but not human cells	[58]
HTLV-1	Tax	Tax SUMOylation and proteasomal degradation	[59]
HTLV-2	APH-2	APH-2 SUMOylation and proteasomal degradation	[60]
ASV-1	IN	Daxx interaction resulting in transcriptional silencing of proviral DNA	[61]

ZIKV: Zika virus; DENV: Dengue virus; HCV: Hepatitis C virus; PV: Poliovirus; EV71: Enterovirus 71; EMCV: Encephalomyocarditis virus; IAV: Influenza A virus; RSV: Respiratory syncitial virus; VSV: Vesicular stomatitis virus; RABV: Rabies virus; LCMV: Lymphocytic choriomeningitis virus; LASV: Lassa virus; RV: Rotavirus; ANDV: Andes virus; PUUV: Puumula virus; HTNV: Hantaan virus; TULV: Tula virus; HFV: Human foamy virus; HTLV: Human T-cell lymphotropic virus; HIV: Human Immunodeficiency virus; ASV: Avian sarcoma virus; NS or NSP: Nonstructural protein; C: Core; 3C^pro^: 3C protease; 3D^pol^: 3D polymerase; M1: matrix; NP or N: Nucleoprotein; P: Phosphoprotein; Z: Zinc finger protein; IN: integrase; Tax: transactivator of the pX region; APH-2: antisense protein of HTLV-2; ND: Not determined.

## Data Availability

No new data were created or analyzed in this study. Data sharing is not applicable to this article.
